# Synthesis of Distinct Iron Oxide Nanomaterial Shapes Using Lyotropic Liquid Crystal Solvents

**DOI:** 10.3390/nano7080211

**Published:** 2017-08-02

**Authors:** Seyyed Muhammad Salili, Matthew Worden, Ahlam Nemati, Donald W. Miller, Torsten Hegmann

**Affiliations:** 1 Chemical Physics Interdisciplinary Program, Liquid Crystal Institute, Kent State University, Kent, OH 44242-0001, USA; ssalili@kent.edu (S.M.S.); anemati@kent.edu (A.N.); 2 Department of Chemistry and Biochemistry, Kent State University, Kent, OH 44242-0001, USA; mworden@austin.utexas.edu; 3 Department of Pharmacology and Therapeutics, University of Manitoba, Winnipeg, MB R3E 0T6, Canada; Donald.Miller@umanitoba.ca

**Keywords:** iron oxide nanoparticles, magnetic nanoparticles, nanosheets, nanodiscs, nanoflakes, lyotropic liquid crystals, template syntheses

## Abstract

A room temperature reduction-hydrolysis of Fe(III) precursors such as FeCl_3_ or Fe(acac)_3_ in various lyotropic liquid crystal phases (lamellar, hexagonal columnar, or micellar) formed by a range of ionic or neutral surfactants in H_2_O is shown to be an effective and mild approach for the preparation of iron oxide (IO) nanomaterials with several morphologies (shapes and dimensions), such as extended thin nanosheets with lateral dimensions of several hundred nanometers as well as smaller nanoflakes and nanodiscs in the tens of nanometers size regime. We will discuss the role of the used surfactants and lyotropic liquid crystal phases as well as the shape and size differences depending upon when and how the resulting nanomaterials were isolated from the reaction mixture. The presented synthetic methodology using lyotropic liquid crystal solvents should be widely applicable to several other transition metal oxides for which the described reduction-hydrolysis reaction sequence is a suitable pathway to obtain nanoscale particles.

## 1. Introduction

The effects of surface chemistry and other properties of functionalized iron oxide nanoparticles (IO NPs) on cell uptake are fairly well established. This is best seen in the large number of reviews published on this topic over the past decade [1,2,3,4,5,6,7,8,9,10,11,12,13,14,15,16,17,18,19,20,21,22,23,24,25,26,27]. Especially multifunctional IO NPs (including surface chemistries for targeting, imaging, and drug delivery, among others) have attracted enormous attention. An often-overlooked aspect, however, is the effect that particle morphology (shape) may have on the theranostic (therapeutic + diagnostic) properties of IO NPs. This is unsurprising, as the vast majority of publications deal with very similar core shapes and sizes. The most common synthetic methods for creating functional IO NPs (i.e., the co-precipitation method by Massart [28], and the solvothermal method by Sun [29]) yield quasi-spherical particles or IO cubes in the range of tens of nanometers or smaller. While there are numerous papers that describe methods for creating non-spherical IO nanostructures [30] (particularly wires [23,31], rods [32,33,34,35,36], cubes [37,38,39], and sheets [40]), fewer of those are used or investigated for in-depth (bio)medical research. While there is a wealth of data for T2 MRI contrast enhancement and magnetic hyperthermia use of such IO nanoshapes [41], specific cell uptake [42] in various relevant tissue or cancer cell lines as well as their intracellular behavior have remained, to a large extent, unexplored.

### 1.1. Shape Effects on Particle Translocation and Endocytosis

Vacha and co-workers conducted molecular dynamics simulations that investigated the effect shape may have on the speed and ease with which nanoparticles may undergo endocytosis [43]. The researchers simulated a model set of nanoparticles, including perfectly spherical, cylindrical, and “spherocylinders” (i.e., cylinders with rounded ends) made of theoretical hydrophilic components they term “beads”. Their computational model compares how spherocylindrical particles and spherical particles undergo endocytosis. The model clearly demonstrates that, with all other surface properties equal, the spherocylindrical particle will pass through the cell membrane much more quickly and efficiently than simple spherical particles with a similar size. The researchers explain these findings by citing Helfrich theory of membrane elasticity, which states that the ability of a particle to be encapsulated by a membrane is inversely proportional to its mean curvature. So, while the attractive energy per unit area is the same for each particle (since both have the same percentage of surface ligands), the mean curvature of the spherocylinder is half that of the sphere and it can thus more efficiently pass through the cell membrane.

Nangia et al. modeled particles of various shapes ranging from spheres and cubes to cone and rice-like geometries in order to investigate translocation rates through cell membranes [44]. In these simulations, the particles were composed of gold. The interactions between particles and the modeled cell membranes were due to shells of charge (both positive and negative, depending on the model) on the surface of the particles, rather than any intrinsic properties of gold. As such, the actual particle composition did not affect the results. Unsurprisingly the researchers found that, regardless of particle shape, negative surface charges on the particles inhibited translocation, while translocation rates increased with increasing positive surface charge. When these surface properties were accounted for, the effect of shape on translocation was clear and dramatic. For the maximum assigned charge density, they found that spherical particles had a free energy barrier for translocation of approximately 60 kJ/mol, giving the second lowest barrier and thus the second highest translocation rate. While these results were superior to those calculated for most of the other shapes, the rice-like particles were found to interact and pass through the cell membrane instantaneously—that is, the free energy barrier was effectively zero. The researchers explain that this is due to the anisotropic shape of the rice-like particles. 

Moving away from computational models, Zhang et al. conducted hands-on experiments in which they compared cell uptake of polystyrene nanospheres with two-dimensional “nanodiscs” [45]. They synthesized both kinds of particles with diameters on the long axis of roughly 20 nm, but the discs were constrained to 2–3 nm along the perpendicular axis. The particles were then compared in cell permeation studies on HeLa cells (human cervical cancer cell line). The researchers found that the nanodiscs preferentially associated with the cell membrane, rarely passing into the cell endoplasm. This was in stark contrast to the nanospheres, which entered into the cells without accumulating along the membrane. They concluded from this that the nanodiscs were retained on the membrane at an 8-fold higher ratio as compared with the nanospheres. These data suggest that these differences are due to the fact that the 2-dimensionality of the nanodiscs allows them to enter into and between the phospholipid bilayers that compose the cell membrane. The nanospheres, which are too large to be maintained between the bilayers, disrupt the membrane to a much greater degree, leading to endocytosis.

### 1.2. The Influence of Iron Oxide Nanoparticle Shape

**Figure 1:**
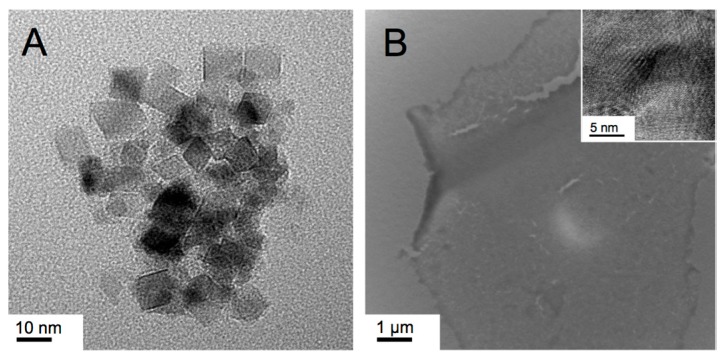
Transmission electron microscopy (TEM) images of previously synthesized IO nanostructures: (**A**) polyhedral nanobricks prepared by co-precipitation of Fe(III)- and Fe(II)-precursors (2:1 ratio) in the LLC phases (lamellar and hexagonal columnar) of Triton X-100 or Triton X-45 in H_2_O [46]. Using TEM tomography the height of the nanobricks was determined to be ~5 nm. Reproduced from Ref. [46] with permission from The Royal Society of Chemistry; (**B**) Large nanosheets (lateral dimensions up to several micron) prepared in the Col_h_ LLC phase formed by Triton X-100 in water using the reduction/hydrolysis method feature heights of 5 or 10 nm (inset shows higher resolution) [47]. Reproduced from Ref. [47] with permission from The Royal Society of Chemistry.

These earlier results on polystyrene nanoshapes are in stark contrast to our own data on IO nanospheres and nanobricks [42]. We examined the influence of IO NP shape in regulating preferential uptake specifically in endothelial cells (brain, lung). NPs targeting endothelial cells to treat diseases such as cancer, oxidative stress, and inflammation have traditionally relied on ligand-receptor based delivery. Spherical (diameter: 10 nm) and brick-shaped IO NPs (polyhedral bricks with 10 nm × 15 nm × 5 nm; *w* × *d* × *h*) were synthesized with identical negatively charged surface EDTS coating (EDTS = *N*-(trimethoxysilylpropyl) ethylenediaminetriacetate trisodium salt; with a ζ-potential around −40 mV). These nanobricks were synthesized using Triton X surfactants in the hexagonal columnar or lamellar lyotropic liquid crystal (LLC) phase (Figure 1A) [46]. The nanobricks showed a significantly greater uptake profile in endothelial cells compared to the IO nanospheres. Furthermore, application of an external magnetic field significantly enhanced the uptake of the nanobricks but not the nanospheres. Transmission electron microscopy (TEM) revealed differential internalization of nanobricks in endothelial cells compared to epithelial cells. Given the reduced uptake of nanobricks in endothelial cells treated with caveolin inhibitors, the increased expression of caveolin-1 in endothelial cells compared to epithelial cells, and the ability of IO NP nanobricks to interfere with caveolae-mediated endocytosis process, a caveolae-mediated pathway was proposed as the working mechanism for the differential internalization of nanobricks in endothelial cells [42]. 

The research briefly discussed above, while clearly intended as a small sampling rather than an exhaustive review, demonstrates that morphology affects how particles interact with cells in profound and important ways. It demonstrates how tuning the shape of particles intended for biomedical applications—IO NPs included—may help us enhance and select certain properties, such the rate of cell uptake or particle interactions with the cells.

A cornerstone of such biomedical studies, particularly for clinically relevant IO nanomaterials, is the development of synthetic methods that create non-spherical IO nanoparticles in a consistent fashion with both shape and size control. Our first synthetic approach made use of a simple, room temperature reduction-hydrolysis of FeCl_3_ in H_2_O/Triton X-100 serving as a lyotropic hexagonal columnar LC template resulting in highly crystalline iron/iron oxide nanosheets [47]. These nanosheets, however, satisfied the requirements for biomedical applications only in one physical dimension, the height of 5 or 10 nm (multiples of 5) as determined by TEM tomography, which was attributed to the coalescence of smaller nanocrystallites to the finally obtained nanosheets in the hexagonal LLC template of Triton X-100 in water with a lattice parameter *d*_10(hex)_ of around 5–6 nm. 

In contrast, the lateral dimensions of the as-obtained I/IO nanosheets were significantly larger, extending in some cases over several hundreds of nanometers (Figure 1B), which is why we tested these for supercapacitor rather than for biomedical applications, where cell uptake, in vivo transport in blood vessels, etc. require much smaller NP dimensions.

Tuning the reaction parameters, i.e., using Massart’s co-precipitation of FeCl_3_ and FeCl_2_ (2:1 molar ratio) [28] in the LLC phases of Triton X-100 (hexagonal columnar phase) or Triton X-45 (lamellar phase) in water, rather than following our in-house developed reduction/hydrolysis pathway [48,49,50,51], resulted in the above-mentioned IO nanobricks with the desirable smaller overall dimensions suitable for endothelial cell uptake, magnetic hyperthermia, as well as MRI contrast enhancement studies. Nevertheless, we were intrigued by the possibility of using various LLC solvents to guide the formation of anisometric IO nanomaterials, and decided to continue to explore this reaction space.

**Figure 2:**
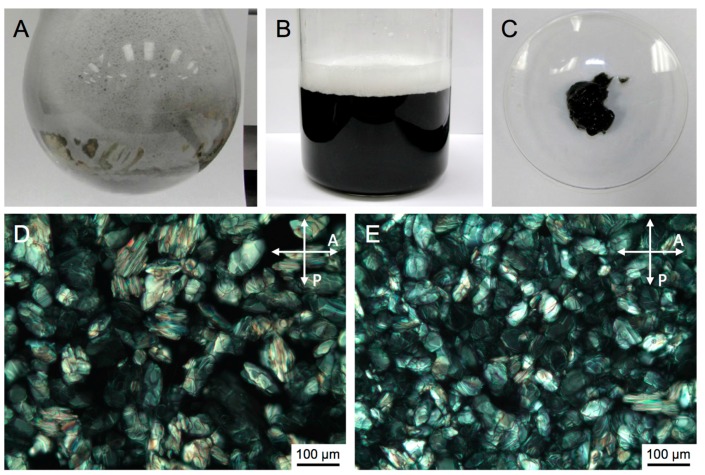
(**A**) Photograph showing the froth formation during after NaBH_4_ addition; (**B**) Photograph showing the diluted froth solution from which as-synthesized nanostructures were isolated by magnetic separation and washing (see experimental section). For the complete experimental setup see photographs in the Supplementary Information; (**C**) Photograph showing the gel fraction that has undergone separate magnetic separation and washing; (**D**,**E**) Polarized light optical photomicrographs (crossed polarizer *P* and analyzer *A*) of the hexagonal columnar phase of one of the used surfactants (here CTAB) in water: (**D**) prior to the addition of the Fe(III)-precursor and (**E**) from a small sample taken during the reaction (The fact that no difference could be detected in the polarized optical microscopy (POM) textures prior to and after addition of the reducing agent indicated the persistence of the Col_hI_ phase as the reaction proceeded).

We demonstrate here that both the type of LLC phase (including micellar solutions of some of the used surfactants in water) and the nature of the surfactant (ionic or neutral) play significant roles in the shape-selective syntheses of IO NPs. Following our reduction/hydrolysis pathway to IO nanomaterials, we also show that various shapes if IO NPs can be synthesized, isolated, and separated as the reactions proceeds. As discussed in our initial article describing this particular synthesis, the formation of H_2_ gas during the reduction of the Fe(III) precursor using NaBH_4_ leads to the formation of two fractions, a gel-like fraction that remains in the reaction vessel and a foam (froth) fraction that is collected separately (Figure 2A–C). We will show that both fractions usually contain dissimilar shapes and sizes of the final IO nanostructures, which are conveniently separated by the synthesis itself.

## 2. Results

Since the originally published, large I/IO nanosheets were the result of a reduction/hydrolysis reaction in the hexagonal columnar phase formed by Triton X-100 in water, we were first interested in testing the reaction in the lamellar (Lα) phase. Since Triton-X-100 in water forms the Lα phase only below 10 °C at higher surfactant concentrations [52], we decided to substitute Triton X-100 with Triton X-45, which forms the Lα phase at room temperature and above over a quite large concentrations range (at least 20 to 60 wt %) [53].

**Figure 3:**
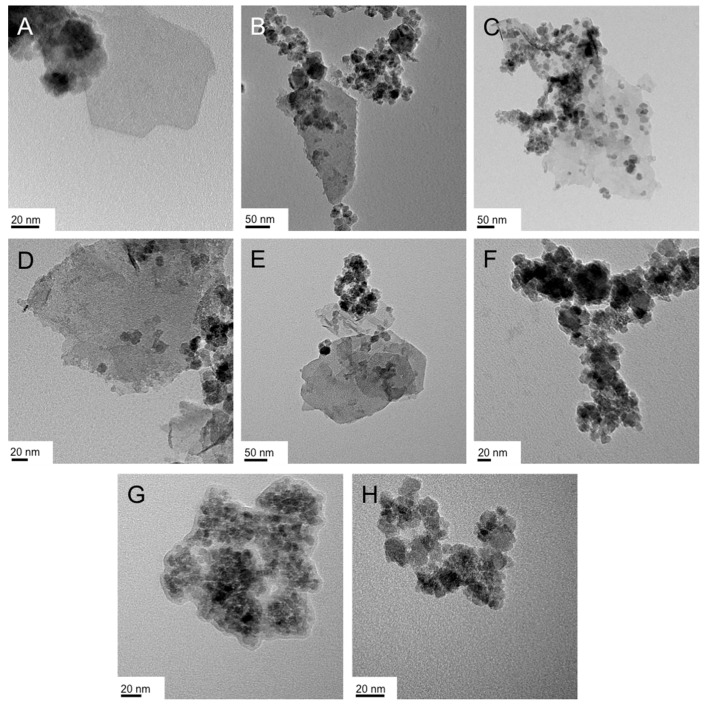
TEM images of the IO nanostructures obtained in the Lα phase of Triton X-45 in water: (**A**,**B**) 50 wt % Triton X-45, 1 mmol FeCl_3_ ((**A**): froth, (**B**): gel); (**C**,**D**) 50 wt % Triton X-45, 2 mmol FeCl_3_ ((**C**): froth, (**D**): gel); (**E**,**F**) 20 wt % Triton X-45, 1 mmol FeCl_3_ ((**E**): froth, (**F**): gel); (**G**,**H**) 50 wt % Triton X-45, 1 mmol Fe(acac)_3_ ((**G**): froth, (**H**): gel).

In numerous instances, running the reaction at room temperature or below proved to be challenging because of the too high viscosities of the bulk LLC phases. For the syntheses with Triton X-45, all reactions were performed at 40 °C. We first varied the concentration of the FeCl_3_·6H_2_O (1 vs. 2 mmol), then the concentration of the surfactant (20 vs. 50 wt %), and finally the type of Fe(III) precursor (chloride vs. acetylacetonate or acac). The TEM images of the representative products are shown in Figure 3. A first, general observation regarding the obtained nanostructures is that the froth fractions (Figure 3A,C,E,G) consist of nanosheets with a varying amount of smaller nanoparticles in the size regime of 15 to 20 nm in diameter. The gel fractions, with the exception of the synthesis with the double amount of FeCl_3_ (2 mmol vs. 1 mmol as in all other cases; Figure 3C,D), contained larger proportions of or even exclusively nanoparticles (Figure 3F,H).

With the original synthesis of extended nanosheets in the hexagonal phase of Triton X-100 in H_2_O [47], we then decided to test if nanosheets would also be obtained in the same surfactant–H_2_O system, but at higher surfactant concentrations (~70 wt %) where Triton X-100 forms an Lα phase, just like Triton X-45 in the syntheses described above. In the case of Triton X-100, the Lα phase is observed only below room temperature up to about 8 °C (see phase diagram by Beyer et al. [52]). 

**Figure 4:**
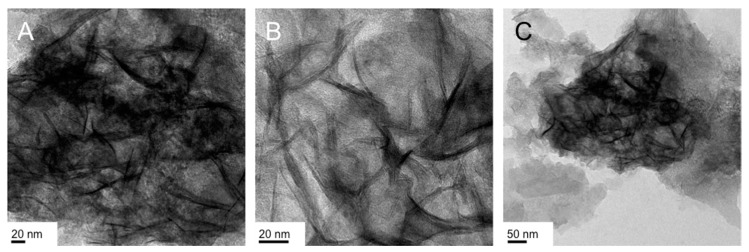
TEM images of the IO nanostructures obtained in the Lα phase of Triton X-100 in water (70 wt % Triton X-100): the reaction was performed at 3 °C and both gel and froth fraction contain the same product (see images in (**A**–**C**)).

The “crumpled” nanosheets (especially those shown in Figure 4A,B) closely resembled the product obtained in the absence of a surfactant described earlier. Significantly smaller sheets, which overlap and appear to fold into each other, can be seen in the representative TEM images shown in Figure 4. Their lateral dimensions rarely exceeded a few hundred nanometers, as can be seen in Figure 4C.

The next surfactant system we investigated was Brij-C10. Brij-C10 in H_2_O gives rise to a variety of LLC phases depending on the surfactant’s concentration. Homogeneous (non-biphasic) LLC phases form above 35 °C, starting with the normal hexagonal columnar (Col_hI_ or H_I_) over the bicontinuous cubic (V_I_ or Cub_I_) to the Lα phase as the concentration of Brij-C10 is raised from about 30 to approximately 80 wt %. As described by others [54], neither the addition of the iron precursors nor the addition of the NaBH_4_ solution leads to a significant change of the overall phase diagram. Starting again with the synthesis in the Lα phase, we first examined two particular concentrations of Brij-C10 in H_2_O (72.5 and 55 wt %). To retain the Lα phase for the lower concentration of Brij-C10 in H_2_O, the temperature for the second synthesis needed to be raised from 62 to 85 °C, since at this concentration the bicontinuous cubic phase below the Lα phase persisted until 78 °C. 

**Figure 5:**
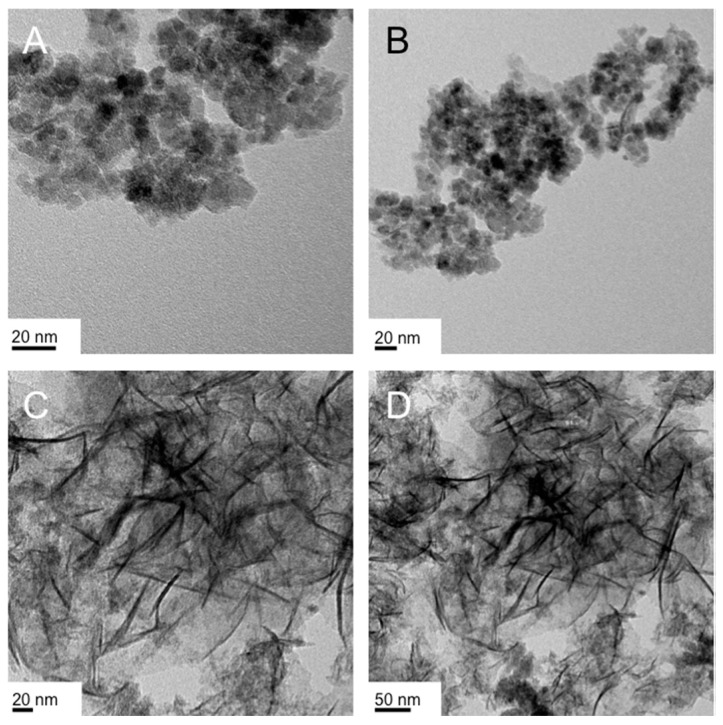
TEM images of the IO nanostructures obtained from the synthesis in the Lα phase formed by Brij-C10 (72.5 wt %) in water at 62 °C: (**A**,**B**) froth fraction and (**C**,**D**) gel fraction.

**Figure 6:**
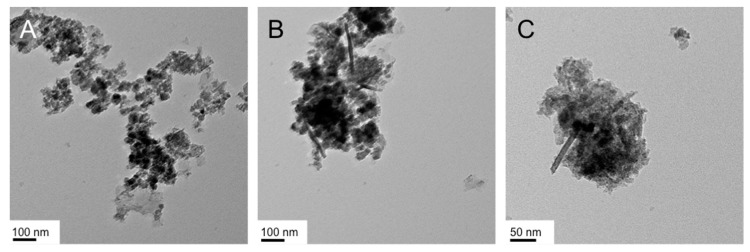
TEM images of the IO nanostructures obtained from the synthesis in the Lα phase formed by Brij-C10 (55 wt %) in water at 85 °C: (**A**,**B**) froth fraction and (**C**) gel fraction.

Figure 5 and Figure 6 show representative TEM images of both syntheses and the characteristic products obtained from both gel and froth fraction. The first key observation when analyzing these two sets of TEM images is that the products (i.e., the morphology of the obtained nanostructures) are very different despite the fact that the differences between both syntheses are only the surfactant concentration and the reaction temperature. The second important observation is that the synthesis at higher surfactant concentration (and lower temperature) produced two entirely different product morphologies, nanoparticles in the size regime of 10 nm in the froth fraction (Figure 5A,B) and the earlier (for Triton X-100 in the Lα phase, Figure 4) described ‘crumpled’ nanosheets (see Figure 5C,D).

At higher temperatures and lower Brij-C10 concentration in the Lα phase, however, froth and gel fractions simultaneously show multiple IO nanostructure morphologies. Smaller flat sheets, even rod-like nanoparticles, and larger polyhedral nanoparticles (up to 40 nm in diameter) can be seen in both the froth and the gel fraction (Figure 6A–C). It appears that at this temperature the reaction is simply too fast to result in products with consistent shape or morphology. 

We also tested the synthesis in the hexagonal columnar phase of Brij-C10 in H_2_O and obtained extended large nanosheets as in the case of Triton X-100 (TEM images similar to those published earlier [47] and shown here in Figure 1B). As expected, the bicontinuous cubic phase was too viscous for the reaction to proceed at all.

To examine the role of the surfactant type (ionic vs. neutral), the final set of syntheses was performed in the micellar solution and hexagonal columnar LLC phase of an ionic surfactant (cetyltrimethylammonium bromide, CTAB) instead of the non-ionic oligoethyleneglycol-based Triton X or Brij series surfactants. CTAB in H_2_O gives rise to a phase diagram similar to Brij-C10 with a normal phase sequence of isotropic (micellar, Iso)–Col_Hi_–Cub_I_–Lα as the concentration of CTAB increases. As shown in the phase diagram reported by Raman et al., at lower concentration of CTAB the shape of the micelles in the two micellar phases changes from quasi-spherical to rod-like, giving rise to two critical micelle concentrations, *CMC*_1_ and *CMC*_2_ [55]. We will initially discuss the two cases of the reduction/hydrolysis reaction in the columnar phase, at first using FeCl_3_ and then the one using Fe(acac)_3_ as iron precursor, each at 1 mmol.

**Figure 7:**
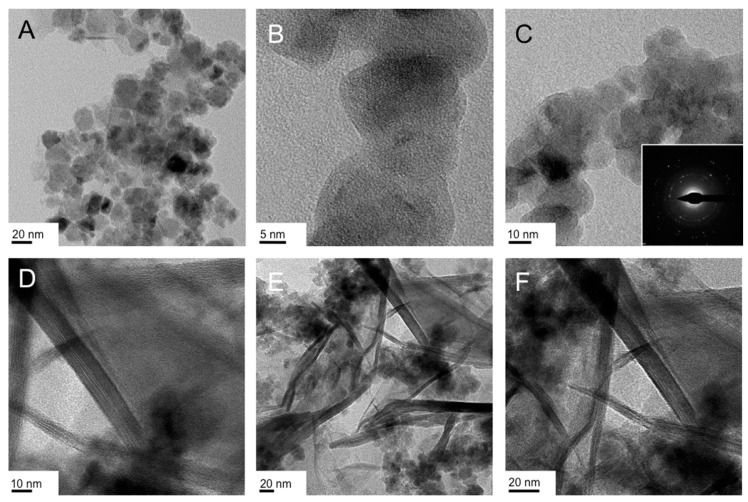
TEM images of the IO nanostructures obtained from the reduction/hydrolysis synthesis of FeCl_3_ in the Col_hI_ phase formed by CTAB (25 wt %) in water at 25 °C: (**A**–**C**) froth fraction and (**D**–**F**) gel fraction. The inset in (**C**) shows the selected area electron diffraction (SAED) pattern, which confirms the crystallinity of these quasi-circular nanodiscs.

We can see right away that the froth (Figure 7A–C) and gel fractions (Figure 7D–F) of the hydrolysis/reduction of FeCl_3_ as iron precursor in the Col_hI_ phase produce entirely different IO nanoscale morphologies. The froth fraction contains what appear to be nanodiscs, some more round others square with rounded edges. The assignment of a disc shape rests on the numerous overlapping and highly crystalline structures for which clear lattice fringes can be seen as, for example, in Figure 7B. The average lateral dimensions of these structures are about 20 nm. The gel fraction, however, contained clearly discernable nanosheets with some smaller nanostructures (somewhat similar to those found in the froth fraction in both size and shape, see Figure 7E). These nanosheets resemble the ‘crumpled’ nanosheets discussed earlier and some are oriented to allow an estimation of the height of these nanosheets to be ~5 or ~10 nm. The lateral dimensions are smaller than those initially obtained for the synthesis in the Col_hI_ phase formed by Triton X-100 in H_2_O [47]. 

Suprisingly, the same reaction at the same conditions now using Fe(acac)_3_ as iron precursor did not have the same outcome with respect to significant differences in shape in the froth and gel fractions.

**Figure 8:**
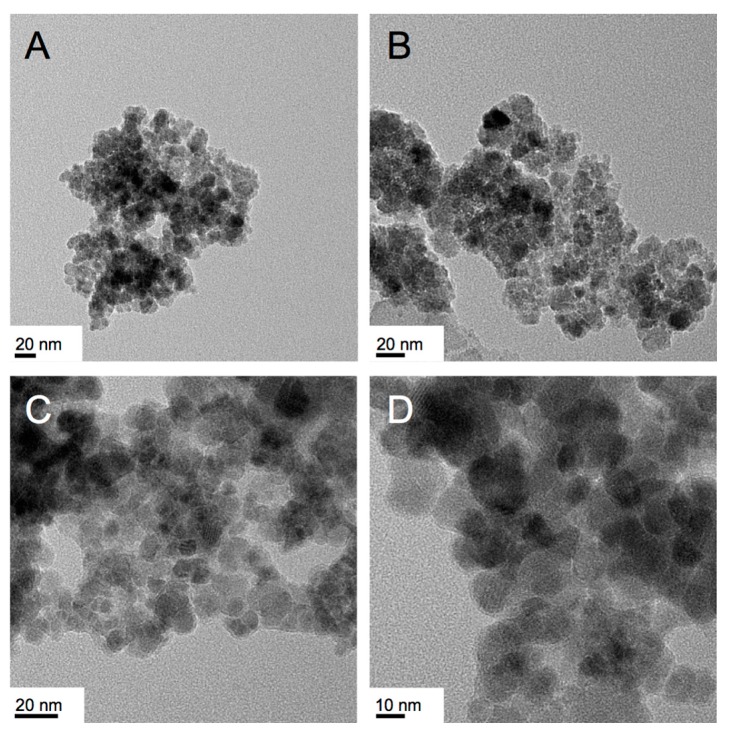
TEM images of the IO nanostructures obtained from the reduction/hydrolysis reaction of Fe(acac)_3_ in the Col_hI_ phase formed by CTAB (25 wt %) in water at 25 °C: (**A**,**B**) froth fraction and (**C**,**D**) gel fraction (lattice fringes are visible for some of the nanostructures in image (**D**)).

As can be seen in the TEM images collected in Figure 8, the froth (Figure 8A,B) contains rough nanoparticle-like structures whose specific shape is not easy to determine from the TEM images. The size of these particles ranges from about 10 to 20 nm. The gel fraction now contained what we previously suggested to be nanodiscs—highly crystalline (lattice fringes are visible at closer inspection), strongly overlapping quasi-spherical shapes with about 10 to 20 nm lateral dimensions (Figure 8C,D). In essence, merely changing the type of iron(III) precuror (or just its counterion) alters two reaction outcomes: (i) what type of morphology is found in froth or gel fraction and (ii) the complete disappearance of nanosheets as a reaction product. Notably, the same also occurred for the related syntheses in the Lα phase of Triton X-45 shown in Figure 3G,H. In fact, the products of that reaction earlier described are very similar to the products of the froth fraction obtained here for CTAB (Figure 8A,B).

Since the Lα phase formed by CTAB in H_2_O only forms at surfactant concentrations above 70 or 80 wt % depending on the temperature (Note: also the Krafft temperature—the temperature at which micelles form—increases markedly for higher concentrations of CTAB in H_2_O), the viscosity of the Lα phase was too high and the solubility of the iron(III) precursors too limited to pursure the reaction. What we did test is the effect of the micelle shape on the reaction outcome by running the reaction in two concentrations of CTAB that form spherical or rod-like micelles (i.e., above *CMC*_1_ and above *CMC*_2_), respectively.

**Figure 9:**
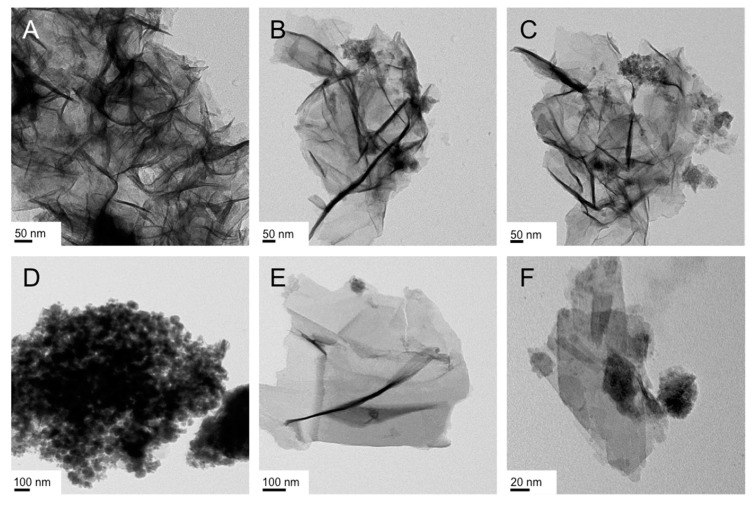
TEM images of the IO nanostructures obtained from the reduction/hydrolysis reaction of FeCl_3_ in the isotropic micellar liquid (spherical micelles) formed by CTAB (3.6 wt %) in water at 50 °C: (**A**–**C**) froth fraction and (**D**–**F**) gel fraction.

At the lower concentration of CTAB in H_2_O (3.6 wt %, i.e., in the aqueous solution featuring spherical micelles) the froth fraction contained, as in several syntheses before, ‘crumpled’ sheets, consistently thin with an average height of about 5 nm (Figure 9A–C). The gel fraction (Figure 9D–F) contained two distinct products with respect to shape, which in some cases could be separated by meticulous washing, magnetic separation and/or centrifugation. As shown in the TEM images, the two shapes are either nanoparticles with a quite significant size distribution (ranging from 10 to 50 nm in diameter) as well as extended nanosheets (Figure 9E) with lateral dimensions of several hundred nanometers. 

**Figure 10:**
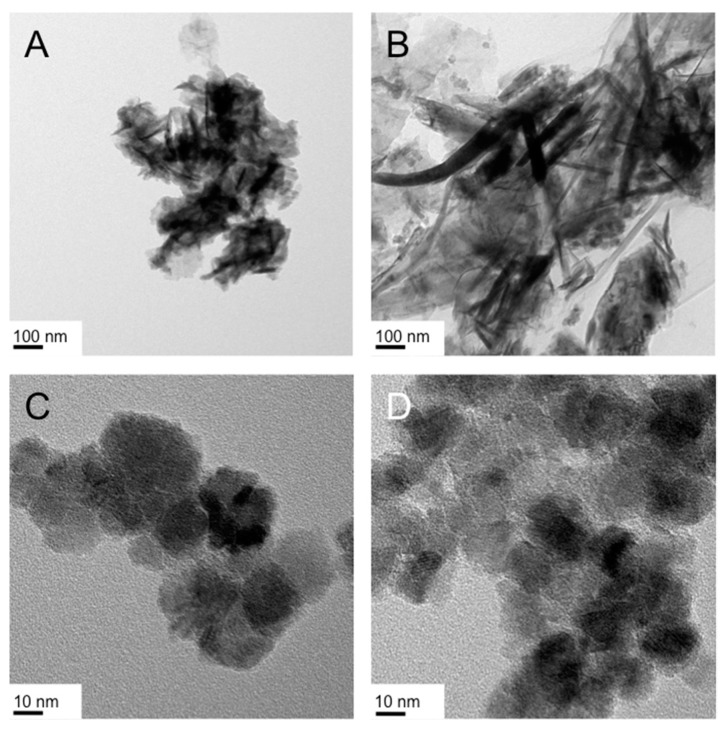
TEM images of the IO nanostructures obtained from the reduction/hydrolysis reaction of FeCl_3_ in the isotropic micellar liquid (rod-like micelles) formed by CTAB (20 wt %) in water at 60 °C: (**A**,**B**) froth fraction and (**C**,**D**) gel fraction.

The same experiment performed in the isotropic phase consisting of rod-like micelles (i.e., at 20 wt % CTAB in H_2_O, above *CMC*_2_) led to the formation of highly “crumpled” nanosheets in the froth fraction (Figure 10A,B) and flower-like nanoparticles in the size regime of 10 to 20 nm in the gel fraction (Figure 10C,D). 

It appears that altering the shape of the micelles in solution (by altering the concentration of the surfactant) affects the nanoscale morphology of the IO nanomaterial shape formed during the reduction/hydrolysis reaction.

**Figure 11:**
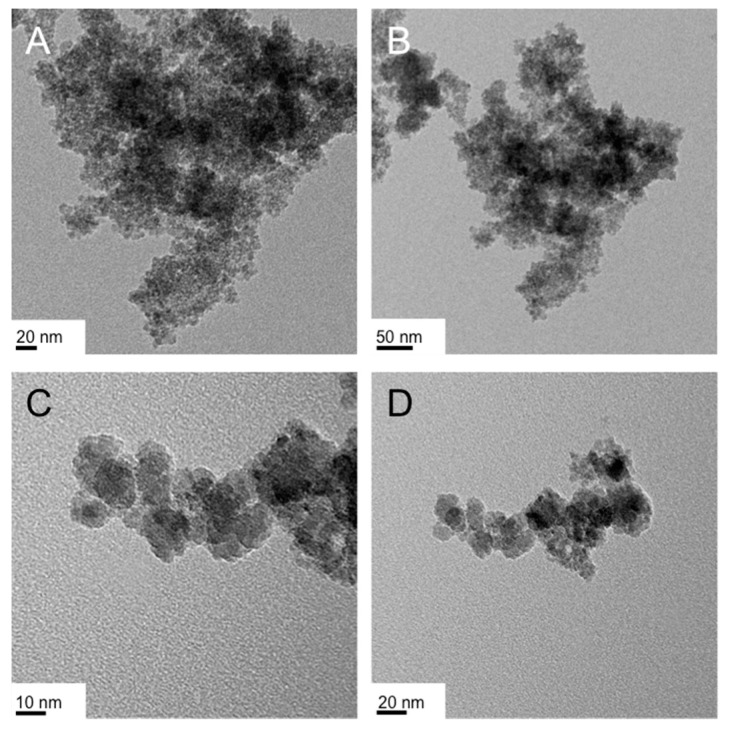
TEM images of the IO nanostructures obtained from the reduction/hydrolysis reaction of Fe(acac)_3_ in the isotropic micellar liquid (rod-like micelles) formed by CTAB (20 wt %) in water at 60 °C: (**A**,**B**) froth fraction and (**C**,**D**) gel fraction.

Finally, exchanging the iron(III) precursor from FeCl_3_ to Fe(acac)_3_ led again to the rough-edged quasi-spherical nanoparticles (Figure 11) obtained earlier from the reduction/hydrolysis reaction of Fe(acac)_3_ in the Col_hI_ phase formed by CTAB in H_2_O (see Figure 8A,B). Considering our original report of this reduction/hydrolysis reaction to obtain pure magnetite (Fe_3_O_4_) nanoparticles using Fe(acac)_3_ as sole iron precursor [51], it appears that the acac ligand, perhaps because of its effect on the iron(III) precursor’s solubility or reactivity, favors the formation of quasi-spherical nanoparticles over flat nanosheet or nanodisc-like morphologies as in the case of iron(III) precursors with chloride counter-ions. The reaction is in both cases incredibly fast (a few minutes), but it is conceivable that acac as bidentate ligand binds to initially-formed nanoparticles, preventing any coalescence to larger, flat sheet- or disc-like nanostructures. 

## 3. Discussion

Table 1 summarizes the size- and shape-dependencies of the IO nanomaterials obtained from the syntheses in the micellar solutions and bulk lyotropic LC phases serving as solvents, depending also on the fraction (froth, gel) from which they were isolated. Particularly astonishing is the fact that even a very subtle change in some of the surfactant systems results in tremendously different IO nanomaterial shapes. For example, doubling the concentration of the iron precursor in the Triton X-45 Lα phase results in nanosheets with larger lateral dimensions, and changing the type of iron precursor brings about significant shape changes (nanosheets vs. NPs in the gel fraction). Changing the type of Triton X surfactant and temperature, but performing the synthesis in both cases in the Lα phase, alters the type of nanosheet (flat and extended vs. crumpled nanosheets). Moving on to another oligoethylene glycol-based surfactant, Brij-C10, and to keep the Lα phase at higher reaction temperatures introduces a more significant amount of polyhedral NPs. This trend appears to be somewhat consistent among the various surfactant systems, in that higher temperatures seem to favor the formation of smaller nanostructures (NPs or nanodiscs) rather than larger extended or crumpled nanosheets. 

The Triton X-100 vs. CTAB syntheses in the Col_hI_ bulk LC phase exemplify the significance of the nature of the surfactant (i.e., neutral vs. ionic): the ionic CTAB typically favors the formation of nanodiscs, which were not observed in the synthesis using Triton X-100. The shape of the micelles in the CTAB isotropic system (above *CMC*_1_ and *CMC*_2_) also shows some effect with no formation of polyhedral nanoparticles in the case of rod-like micelles, unless Fe(acac)_3_ is used as the iron precursor. As a more general observation, for the discussed reduction-hydrolysis reaction, Fe(acac)_3_ in almost all cases favors the formation of polyhedral, quasi-spherical NPs instead of nanosheets or nanodiscs favored when FeCl_3_ is the iron precursor.

This is consistent with our earlier observation of the formation of NPs from Fe(acac)_3_ in our reduction-hydrolysis reaction [51], which might be due to the fact that the reduced form of acac, i.e., pentane-2,4-diol [56], could act as a stabilizing bidendate ligand preventing the coalescence of initially formed nanocrystallites that form the basis of the larger nanosheets formed in the case of FeCl_3_ as iron precursor. As for the mechanism of the formation of nanosheets and nanodiscs, the dimensions of the lamellar sheets in the Lα phases and the rod-like micelles in the Col_hI_ phases appear to play a signficant role.

**Figure 12:**
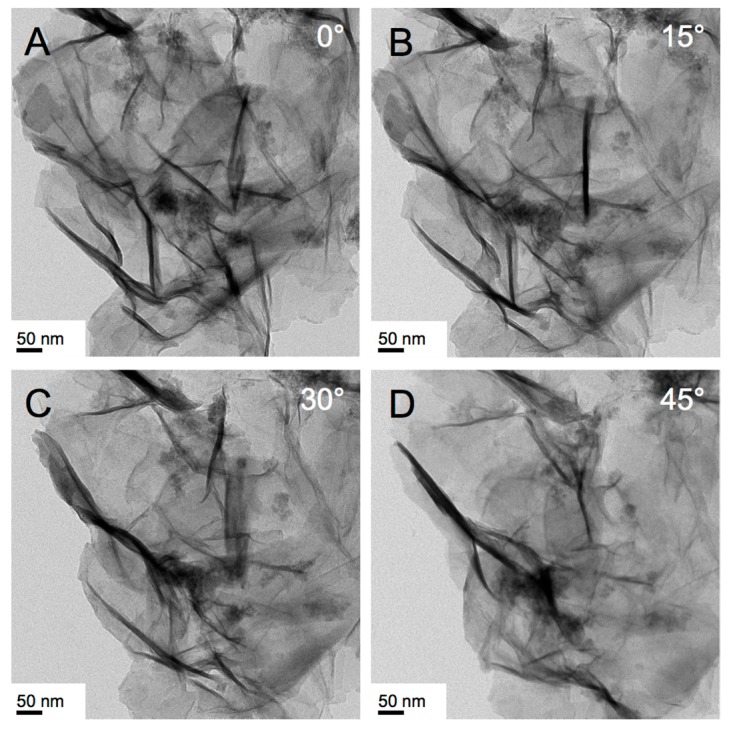
TEM tomography images (various degrees of tilt) of crumpled IO nanosheets obtained via the reduction/hydrolysis reaction of FeCl_3_ in the isotropic micellar liquid (spherical micelles) formed by CTAB (3.6 wt %) in water at 50 °C (froth fraction).

As described before, the height of the flat nanostructures always appears to be a value close to or multiple-times the layer spacing (Lα) or lattice paramater (Col_hI_) of the lyotropic LC phase used as a solvent [47], as determined by TEM tomography (for an example see Figure 12). We therefore argued that confinement in the growth of IO nanostructures within the lamellae or columnar aggregates of the LLC structure, as well as confinement on the gas bubbles forming the froth, play important roles in the formation of the various IO nanostructures [47]. However, as exemplified by the synthesis that uses the Lα phase of Brij-C10 (Figure 5), significant differences in nanostructure morphology in froth and gel fractions cannot solely be explained by confinement on the froth’s gas bubbles. In most other syntheses involving oligoethylene glycol-based surfactant systems (i.e., Triton X-45 and X-100), nanosheets were predominantly formed in the froth and not in the gel fraction. In the case of Brij-C10 (at 72.5 wt % in H_2_O and 62 °C, Figure 5A–D), “crumpled” nanosheets formed exclusively in the gel fraction, perhaps due to the higher viscosity and the resulting lower rate of IO nanostructure formation. Hence, a conclusive general mechanism of IO nanostructure formation in these LLC solvents is rather difficult to deduce considering additional effects such as coordination of atoms or groups of atoms of the various surfactant molecules to the IO nanostructure surfaces (e.g., oxygen atoms in the oligoethylene glycol segments) and differences in viscosity that affect the rate of IO nanostructure formation. The viscosity of a given LLC solvent system is governed by various parameters such as temperature, surfactant and precursor concentration, as well as the type of micellar or LLC phase. In this sense, the LLC phases are not simply passive templates but rather complex anisometric as well as coordinating solvents that affect both shape and dimensions of the formed IO nanomaterials in very specific ways depending on all synthesis parameters.

Considering earlier data on the differential cell internalization of polyhedral IO nanobricks vs. quasi-spherical IO NPs [42], future investigations of cell uptake by endothelial as well as epithelial cells will focus particularly on the smaller nanodiscs obtained from several of the CTAB–H_2_O LLC solvent systems. These comparative studies will allow us to expand our understanding of the role of NP shape in regulating preferential uptake of NPs over a wider range of IO nanomaterial shapes, including nanospheres, -bricks, and -discs.

## 4. Materials and Methods

Iron(III) chloride hexahydrate (FeCl_3_·6H_2_O, 98%), iron(III) acetylacetonate (Fe(acac)_3_, 97%,), sodium borohydride (NaBH_4_, 98%), cetyltrimethylammonium bromide (CTAB, ≥99%), Triton™ X-100 (laboratory grade), and Triton™ X-45, Brij^®^ C10 were purchased from Sigma-Aldrich (St. Louis, MO, US)and used as such for synthesis. Deionized (DI) water (*R* = 18 MΩ) was used in all reaction and purification steps. Transmission electron microscopy (TEM) was carried out using a FEI Tecnai TF20 TEM instrument (Hillsboro, OR, USA) at an accelerating voltage of 200 kV. Particle samples were dispersed in water or methanol and a droplet was placed onto a carbon coated copper grid (400 mesh) and finally air-dried prior to analysis. Polarized Optical Microscopy (POM) studies were carried out using an Olympus BX-53 microscope equipped with a Linkam LTS420E (Waterfield, UK) heating/cooling stage. For each reaction, small samples were taken before and after the addition of the reducing agent (NaBH_4_), and the LLC phase formed and retained was confirmed by careful texture observations between crossed polarizers (for an example, see Figure 2D,E). 

The surfactant weighed into the reaction flask was degassed under high vacuum for two hours. Separately, FeCl_3_·6H_2_O (1 or 2 mmol) or alternatively Fe(acac)_3_ in the appropriate amount of DI water (to obtain the LLC phase or micellar solution of interest to a total of about 50 mL) was purged with nitrogen for one hour. Thereafter, the FeCl_3_ or Fe(acac)_3_ solution was added to the surfactant in the reaction flask under a steady flow of nitrogen, which was maintained until the completion of the reaction. The contents were mixed using a mechanical stirrer at 100 RPM, until the mixture turned to a taffy-like consistency. At this stage, the contents were slowly warmed to 40 or 50 °C (until all solids went into solution to form the micellar solution or bulk LLC phase) and cooled back to ambient temperature to form a yellow-colored bulk LLC phase (checked by POM). NaBH_4_ (10 or 20 mmol) in 5 or 10 mL deoxygenated DI water was added rapidly to the reaction vessel with occasional mechanical stirring. The amount of water to prepare the NaBH_4_ solution was taken into account when calculating the surfactant concentrations to obtain specific micellar solutions or LLC phases. The yellow gel turned almost immediately to a black color along with formation of excess froth that filled the reaction vessel. Although the reaction is complete after approximately two minutes, after an additional 30 min from adding NaBH_4_, the black-colored froth was collected by adding 200 mL DI water in portions, leaving the gel part behind. The crude product from the froth was collected using a rare earth magnet and washed several times with DI water and followed by ethanol. The final product after washing was dried at room temperature in air to yield a black powder. The product isolated from the gel portion after washing with copious amounts of water followed by ethanol was a dark blackish powder. This synthesis procedure was followed for all surfactants and surfactant concentrations—amounts, ratios, and concentrations are given in the captions of the TEM images. 

## 5. Conclusions

We have shown that surfactant-water systems, both as bulk lyotropic LC or micellar solutions, are noteworthy solvents for the shape- and size-controlled synthesis of IO nanostructures. Using the reduction-hydrolysis reaction of a single iron(III) precursor discovered in our laboratory we were able to synthesize a range of magnetic IO nanostructures with disc, sheet, crumpled sheet, as well as polyhedral shapes. Based on the datasets gathered from TEM image analysis we were also able to establish how parameters such as type and concentration of the iron(III) precursor, LLC phase, type of surfactant, and temperature affect the synthesis outcome, especially with respect to the shape of the IO nanostructures. We believe that these results bode well for continued studies on template-based syntheses of metal and metal oxide nanomaterials using LLC phases. As outlined in the introduction, especially for IO nanomaterials, shape is of critical importance for differential cell uptake, hyperthermia, MRI contrast enhancement, and drug delivery. For metal oxides in general, the large range of possible, already considered and tested applications aside from medical uses will certainly benefit from synthetic tools that allow for control over nanomaterial size and shape. 

## Figures and Tables

**Table 1:** Summary of nanostructures formed in the various micellar solutions and LLC phases.

Surfactant	Iron Precursor/Conc., Phase/wt % Surfactant, Temperature ^1^	Main Morphology ^2^ Gel [Froth]	Dimensions ^3^ *w* × *d* × *h* (nm)
Triton X-45	FeCl_3_/1 mmol, Lα/50, 40 °C	NS [NS + NPs]	100 × 100 × 5 [20]
	FeCl_3_/2 mmol, Lα/50, 40 °C	NS + NPs [NS + NPs]	300 × 300 × 10 [20]
	FeCl_3_/1 mmol, Lα/20, 40 °C	NP [NS]	300 × 300 × 10 [20]
	Fe(acac)_3_/1 mmol, Lα/50, 40 °C	NP [NP]	15–20 [15–20]
Triton X-100	FeCl_3_/1 mmol, Col_hI_/48, 25 °C [47]	NS [NS]	500+ × 500+ × 5, 10
	FeCl_3_/1 mmol, Lα/70, 3 °C	NS_cr_ [NS_cr_]	100+ × 100+ × 5, 10
Brij-C10	FeCl_3_/1 mmol, Lα/72.5, 62 °C	NS_cr_ [NP]	10 [100+ × 100+ × 5, 10]
	FeCl_3_/1 mmol, Lα/55, 85 °C	NP + few NS_cr_ [NP]	40 [40]
CTAB	FeCl_3_/1 mmol, Col_hI_/25, 25 °C	NS + NP [ND]	100 × 100 × 10 [20] ^4^
	Fe(acac)_3_/1 mmol, Col_hI_/25, 25 °C	ND [NP]	20 ^4^ [20]
	FeCl_3_/1 mmol, Iso_sp-mic_/3.6, 50 °C	NS + NP [NS_cr_]	500+ × 500+ × 10, 20
	FeCl_3_/1 mmol, Iso_rl-mic_/20, 60 °C	ND [NS_cr_]	20–30 ^4^ [100+ × 100+ × 20]
	Fe(acac)_3_/1 mmol, Iso_rl-mic_/20, 60 °C	NP [NP]	10–20 [10–20]

^1^ Parameter adjusted in bold font; ^2^ NS = nanosheet, NS_cr_ = “crumpled” NS, NP = nanoparticle, ND = nanodisc, Iso_sp-mic_ = isotropic liquid containing spherical micelles, and Iso_rl-mic_ = isotropic liquid containing rod-like micelles; ^3^ Where applicable for polyhedral shapes; otherwise the average diameter of the polyhedral NPs is given. ^4^ The diameter of the ND is given.

## Supplementary Materials

The following are available online at http://www.mdpi.com/2079-4991/7/8/211/s1.
