# Supplementary material for: Synthesis of Distinct Iron Oxide Nanomaterial Shapes Using Lyotropic Liquid Crystal Solvents
Source: Nanomaterials (Basel). 2017 Aug 2;7(8):211. doi: 10.3390/nano7080211 (PMC5575693; doi:10.3390/nano7080211)
Supplement: Supplementary file 1 [file nanomaterials-07-00211-s001.pdf]

Supplementary Information

for

Synthesis of Distinct Iron Oxide Nanomaterial Shapes using Lyotropic Liquid Crystal Solvents

by

*Seyyed Muhammad Salili, Matthew Worden, Ahlam Nemati, Donald W. Miller and Torsten Hegmann**

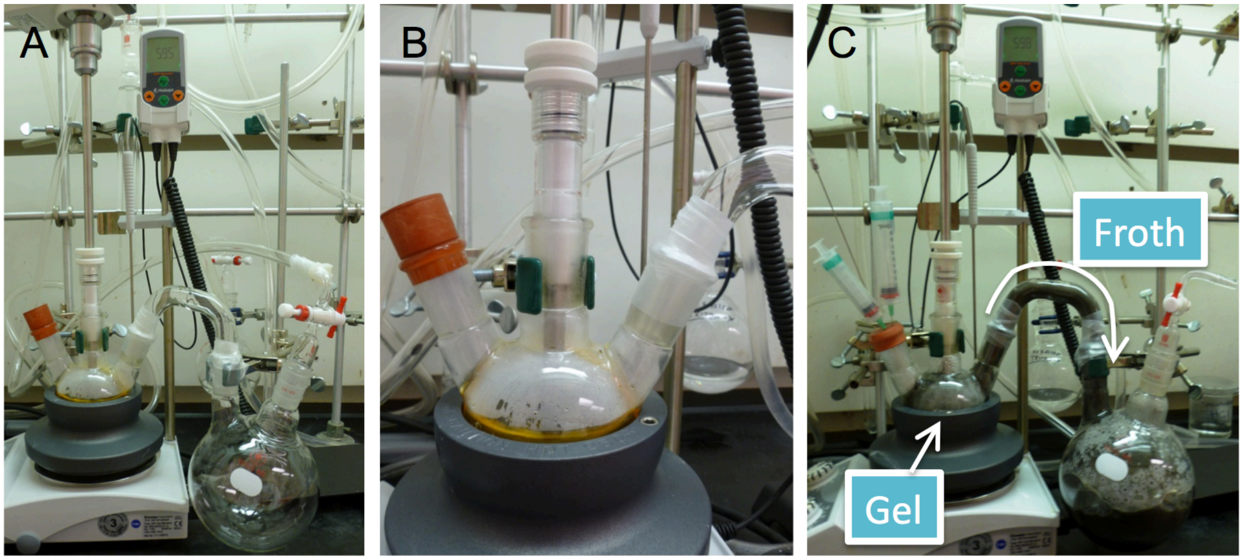

Figure S1. Photographs (A) and (B) show the reaction setup and (C) the reaction progress: (A, B) bulk LLC phase charged with Fe(III) precursor solution, (C) after injection of $NaBH_4$ solution. The formation of IO nanomaterials is almost instantaneous as indicated by the color change and the formation of froth that is collected in a separate flask.
